# Influence of Ecological Factors on the Metabolomic Composition of Fish Lenses

**DOI:** 10.3390/biology11121709

**Published:** 2022-11-25

**Authors:** Yuri P. Tsentalovich, Ekaterina A. Zelentsova, Ekaterina D. Savina, Vadim V. Yanshole, Renad Z. Sagdeev

**Affiliations:** International Tomography Center SB RAS, Institutskaya 3a, Novosibirsk 630090, Russia

**Keywords:** freshwater fish, metabolomics, dissolved oxygen level, water pollution, NMR spectroscopy

## Abstract

**Simple Summary:**

Biological responses to rugged ecological factors causes changes in the metabolic pathways in aquatic organisms, which may manifest as changes in the tissue metabolomic composition. Therefore, establishing a correlation between the metabolite concentrations in aquatic animals and ecological factors is important for understanding the molecular mechanisms of biological responses to ecological stresses. In this work, we determined the concentrations of 57 major metabolites in the lenses of fish from three locations in Siberia (Russia) that differed in levels of dissolved oxygen (LDO) and water purity. We found that the increased due to CO_2_ emissions water acidity and the reduced LDO caused significant changes in the fish lenses’ metabolomic compositions, including amino acids, organic acids, and energy metabolites. The obtained results can be used in monitoring the ecological state of water bodies.

**Abstract:**

Multiple stressors related to changes in environmental conditions (such as water temperature, salinity, and natural and anthropogenic pollution) may cause biological responses of aquatic organisms that lead to significant variations in the biochemical reactions in their tissues and thereby change the concentrations of metabolites. We used a quantitative NMR-based metabolomic analysis of the fish lens for the evaluation of the influence of environmental factors on metabolic processes in aquatic animals. For this purpose, three species of freshwater fish—Perca fluviatilis, Rutilus rutilus lacustris, and Gymnocephalus cernua—were caught at approximately the same time at three locations in Siberia (Russia) that differed in levels of dissolved oxygen (LDO) and water purity, and the concentrations of 57 major metabolites in the fish lenses were determined. We found that the metabolomic profiles of the fish lenses strongly depended on the location. The obtained data demonstrated that two typical stressors for aquatic animals—a reduced LDO and anthropogenic water pollution—caused a largely similar metabolic response in the fish lenses that led to an increase in the concentrations of several amino acids and a decrease in sarcosine and phosphoethanolamine. At the same time, the composition of the major lens osmolytes depended mostly on the oxygen level, while variations in AMP (decrease) and NAD (increase) corresponded to the water pollution. We suggest that the eye lens is a very convenient tissue for studying the impact of ecological factors on the metabolic state of aquatic animals, fish in particular.

## 1. Introduction

The metabolomic analysis of wild animal tissues is a promising tool for monitoring the influence of environmental factors on animal health [1]. Metabolomic studies are especially important for aquaculture because variations in the external parameters such as the water temperature and salinity [2,3,4,5,6,7], level of dissolved oxygen [8,9,10,11,12], and water body pollution [13,14,15,16,17,18,19,20] can have a significant effect on the metabolomic composition of fish and other marine and freshwater animals and can cause the development of various diseases. Very often, samples of muscle from fish or other animals are subjected to metabolomic analyses because this tissue is the most used in food production [21,22]. However, the metabolomic composition of metabolically active tissues such as muscle or liver may experience significant short-term and sample-to-sample variations [23]. The eye lens, being a more conservative and anatomically isolated tissue, might be more suitable for the study of the impact of external factors on fish health [24]. The influx of fresh metabolites into the lens and the removal of metabolic waste proceed through the aqueous humor (AH), which, in turn, is connected with blood via the ciliary epithelium and Schlemm’s canal. The AH provides the metabolite flux into and out of the lens. The metabolomic composition of the AH is very similar to that of blood [25]; because the lens cells possess minimal metabolic activity, the composition of the majority of the metabolites in the lens reflects a metabolomic profile of the whole body that is smoothed in time. As the result, the sample-to-sample data spread for lenses is lower than for other tissues, which makes it possible to determine statistically significant metabolomic changes within the smaller groups [23]. At the same time, a number of important compounds, including antioxidants, osmolytes, and energy metabolites, are synthesized inside the lens, and variations in the concentrations of these metabolites indicate the lens-specific cellular responses to the changes in external factors.

In our recent study [26], we showed that the metabolomic composition of the lenses of freshwater fish underwent significant seasonal variations in the period from the late autumn to the early spring. The most pronounced changes were observed for intracellular osmolytes (*myo*-inositol, N-acetyl-histidine (NAH), N-acetyl-aspartate (NAA), threonine–phosphoethanolamine (Thr-PETA), and serine–phosphoethanolamine (Ser-PETA)), antioxidant ovothiol A (OSH), and energy metabolites (ATP, ADP, and NAD). These changes were attributed to a decrease in the dissolved oxygen level (LDO) in ice-covered lakes due to an impeded gas exchange through the water–air interface [27], low feeding activity, and a deceleration of the metabolic processes in the fish during the winter.

One of the challenges in metabolomic studies is that different environmental factors may cause similar biological responses and similar changes in the metabolomic compositions of animal tissues. For example, changes in the energy metabolism that lead to an increase in the lactate concentration can be caused by hypoxia [8,11,28,29], water pollution [18,19,30], and the water temperature [4]. On the other hand, the same stressor may cause different metabolic responses depending on the species, tissue, and time of exposure [31]. Moreover, several environmental factors may act simultaneously; in field studies, it is often difficult to separate the contributions of different factors into observed changes in the metabolomic profiles. In the present work, we attempted to separate the impacts of a reduced LDO and water pollution from other environmental factors. To this end, we analyzed the quantitative metabolomic compositions of fish lenses from three species with different feeding habits: the predatory European perch (*Perca fluviatilis*), the herbivorous Siberian roach (*Rutilus rutilus lacustris*), and the omnivorous Eurasian ruffe (*Gymnocephalus cernua*). The fish were caught at approximately the same time (March 2019) at three places corresponding to the Ob River basin (Siberia, Russia): the Nigiya River, Lake Borovoye, and the Ob Reservoir (Figure S1). All three locations are relatively close to each other and have similar seasonal conditions; however, the water bodies’ physicochemical and biochemical properties differ. The goal of this study was to estimate the influence of water properties on the metabolome of the fish eye lens.

## 2. Materials and Methods

### 2.1. Locations

The Nigiya River (59°10’40” N, 81°47’18” E) is a tributary of the Ob River in the Tomsk region (Siberia, Russia); it flows through an ecologically clean area in a taiga with a very low population density and minimal industrial activity. The river originates from peat bogs; the water contains a high amount of organic matter. Even during the winter, the water in the river is turbid and has a distinct greenish color. In the winter, the river is mostly covered by ice, but due to the fast water movement, some small parts of the river remain uncovered through the entire winter; nevertheless, the biological activity of algae and the decay of organic material lead to a rather low LDO. During the expedition (March 2019), the measured LDO was 6.8 ± 0.3 mg/L (Table 1). The Nigiya River is directly connected to the Ob River; the fish population includes a broad spectrum of herbivorous and predatory fish.

Lake Borovoye (59°8’4” N, 81°47’42” E) is located approximately 10 km away from the Nigiya River. The lake is isolated from the Ob River and has a round shape with a diameter of 1 km; the depth of the lake does not exceed 5–6 m. The water in the lake is very clean. It is fed by underground springs that provide a very high LDO of 13.1 ± 0.3 mg/L even in March, although the lake is completely covered by ice. The major fish species in Lake Borovoye are the predators pike and perch.

The Ob Reservoir (54°50’12” N, 83°01’53” E), an artificial basin on the Ob River located near the 1.5-million-resident Novosibirsk megalopolis, is inhabited by a large variety of fish species. The pollution of the Ob Reservoir by industrial and agricultural wastes originates from Novosibirsk itself and cities located along the Ob Reservoir. The Ob Reservoir is covered by ice in March, but due to the large sizes of the Ob River and the Ob Reservoir (more than 1000 km^2^), the LDO in winter remains relatively high; the measured LDO was 9.7 ± 0.3 mg/L.

The distance between the Ob Reservoir and the two other locations is approximately 500 km (Figure S1), but the climatic conditions at all three locations are very similar. In particular, ice freezes in the Novosibirsk and Tomsk regions in the second half of November, while the ice-breaking occurs in the end of April. During the sample collection (March 2019), the water temperature was 4–5 °C at all three locations.

### 2.2. Measurements of LDO

The LDO was measured with the use of an HQ30d HQD meter (Hach Lange GmbH, Dusseldorf, Germany) equipped with a LDO101 electrode. The measurements were performed at the coordinates shown in the “Locations” section through holes drilled in the ice. During the measurements, the electrode was immersed in water below the ice level. For every location, the measurements were performed for three holes, and for every hole the LDO was measured three times with an interval of 5–10 min between measurements. The averaged data are given in Table 1.

### 2.3. Biochemical Analysis of Water Samples

The samples of water taken at the fishing locations were placed in plastic containers (3 L from each location), frozen, and then transported to the laboratory. The samples were kept at −70 °C until analysis. The biochemical analysis of the water samples was performed at the “ESG Occupational Safety and Health Ltd.” testing laboratory, Moscow, Russia (accredited for water analysis under accreditation certificate RU.0001.519176). The results of the analysis are shown in Table 1.

### 2.4. Lens Sample Collection

The study was conducted in accordance with the ARVO Statement for the Use of Animals in Ophthalmic and Vision Research and the European Union Directive 2010/63/EU on the protection of animals used for scientific purposes, as well as with the ethical approval of the International Tomography Center (ECITC-2017-02). No special permission from the national or local authorities was required.

The sample collection was performed in March 2019; i.e., approximately one month before the ice-breaking. In all cases, the fish were caught with the use of a winter fishing rod through holes drilled in the ice. Three species of fish—*P. fluviatilis*, *R. rutilus lacustris*, and *G. cernua*—were caught in the Nigiya River and in the Ob Reservoir, while only one species—*P. fluviatilis*, in Lake Borovoye. The fish were of both sexes, and their sizes varied between 120 and 200 g for *P. fluviatilis*, 70 and 120 g for *R. rutilus lacustris*, and 30 and 50 g for *G. cernua*. The fish were euthanized with a concussive blow to the head and the lenses were cut from the fish immediately after being caught; the lenses were then placed in 2.0 mL plastic vials and frozen. At each location, we collected one lens from each of five individuals of the same species. The frozen samples were transported to the laboratory and kept at −70 °C until analysis. The characterization of the fish lenses used in this work is given in Supplementary Table S1.

### 2.5. Sample Preparation

The preparation of the lens samples for the metabolomic analysis was performed as described in [26]. Each fish lens was weighted and homogenized with a TissueRuptor II homogenizer (Qiagen, Venlo, Netherlands) in 1600 µL of cold (−20 °C) MeOH, and then 1600 µL of cold chloroform (−20 °C) and 800 µL of water were added. The mixture was shaken in a shaker for 20 min and left at −20 °C for 30 min. Then, the mixture was centrifuged at 16,100× *g* at +4 °C for 30 min, which yielded two immiscible liquid layers separated by a lipid–protein layer. We collected and lyophilized the upper aqueous layer (MeOH-H_2_O).

### 2.6. NMR Measurements

We re-dissolved dry protein-free lipid-free extracts in 600 μL of 20 mM deuterated phosphate buffer (pH 7.2) containing 2 × 10^−5^ M sodium 4,4-dimethyl-4-silapentane-1-sulfonic acid (DSS) as an internal standard. The ^1^H NMR spectra were obtained using an AVANCE III HD 700 MHz NMR spectrometer (Bruker BioSpin, Ettlingen, Germany) equipped with a 16.44 Tesla Ascend cryomagnet at the Center of Collective Use “Mass spectrometric investigations” SB RAS as described in [26].

### 2.7. NMR Signal Identification and Metabolite Quantification

We identified the metabolites in the NMR spectra according to their NMR spectra available in the literature [32] and in our in-house library [23,25,26,33,34]. In questionable cases, signal attribution was confirmed by spiking the extract with commercial standard compounds. The obtained NMR spectra of the lens extracts and the signal assignment were very similar to spectra published earlier [26]. The baseline correction and integration were done manually using the program MestReNova v12.0 (Mestrelab Research, A Coruna, Spain). We determined the metabolite concentrations in the extracts according to the NMR signal area integration relative to the internal standard DSS followed by the calculation of the metabolite concentrations in a tissue (in nmoles per gram of the sample wet weight). The list of quantified metabolites, their chemical shifts, and their multiplicity are provided in Table S2.

### 2.8. Data Analysis

The chemometric data analysis, including a principal component analysis (PCA) and Metabolite Set Enrichment Analysis (MSEA), was performed on a MetaboAnalyst 5.0 web platform (www.metaboanalyst.ca [35], accessed on 23 February 2022). The PCA scores and loadings plots were constructed using autoscaling of the data (mean-centered and divided by the standard deviation of each metabolite concentration) to normalize the contributions of all metabolites. The quantitative MSEA was performed for quantitative data without prior normalization with the Global test algorithm using the SMPDB pathway library from the small molecule pathway database accounting for 99 pathways. Two group pairs were analyzed using MSEA: *P. fluviatilis* lenses were compared for Borovoye vs. Nigiya (difference in LDO) and Borovoye vs. Ob (anthropogenic water pollution).

## 3. Results

In this work, a quantitative metabolomic analysis was performed using lenses of *P. fluviatilis*, *R. rutilus lacustris*, and *G. cernua* caught in March 2019 at three locations: the Nigiya River, Lake Borovoye, and the Ob Reservoir. We determined the concentrations of 57 major metabolites including amino acids, organic acids, osmolytes, antioxidants, sugars, nucleotides, and nucleosides (Table S2). The obtained results are presented in Table S3; the compound concentrations are expressed in units of nmoles per gram of wet tissue and given as the mean ± std (*n* = 5). 

We measured the physicochemical and biochemical properties of water samples taken from three locations. The list of monitored parameters is provided in Table 1. Among all parameters, the LDO and water pH level could be selected as the most important ones because the levels of the majority of the other potential stressors were well below the limits of permissible concentrations. A high water acidity reflected the elevated level of CO_2_ caused by industrial emissions (anthropogenic pollution): carbon dioxide dissolved in water forms carbonic acid and causes the pH to decrease. The LDO depended on several natural and anthropogenic factors, and it varied significantly between the locations under study.

The following general characterizations can be given to the basins related to the present study:

The water samples from the Nigiya River featured elevated levels of ammonium ion, iron, chemical and biological oxygen demands, suspended substances, and permanganate oxidizability (Table 1). All other parameters were below the values of maximum permissible concentrations for surface waters. Therefore, we assumed that the decreased oxygen level was the main stressor produced by the decay of natural organic matter, while the levels of other chemical pollutants were low. The basin had a neutral pH 6.8 (Table 1), which indicated low anthropogenic pollution by CO_2_ emissions (code OlPl).

Lake Borovoye had a high LDO and neutral pH of 7.5 (Table 1), which indicated low CO_2_ pollution (code OhPl).

The Ob Reservoir’s LDO measurements demonstrated that although the oxygen level in this basin was lower than that in Lake Borovoye, it was still relatively high. The contents of manganese and nitrate were noticeably higher (two orders of magnitude) than those in the Nigiya River and Lake Borovoye, although they remained within the levels permitted by the authorities’ limits for surface waters (Table 1). The only parameter that exceeded the maximum permissible concentration was the elevated water acidity (pH 5.3) caused by Novosibirsk’s industrial emissions. Thus, in this work we considered the Ob Reservoir as the water body with a moderate LDO and a moderate level of anthropogenic pollution by CO_2_ emissions (code OmPm).

**Figure 1:**
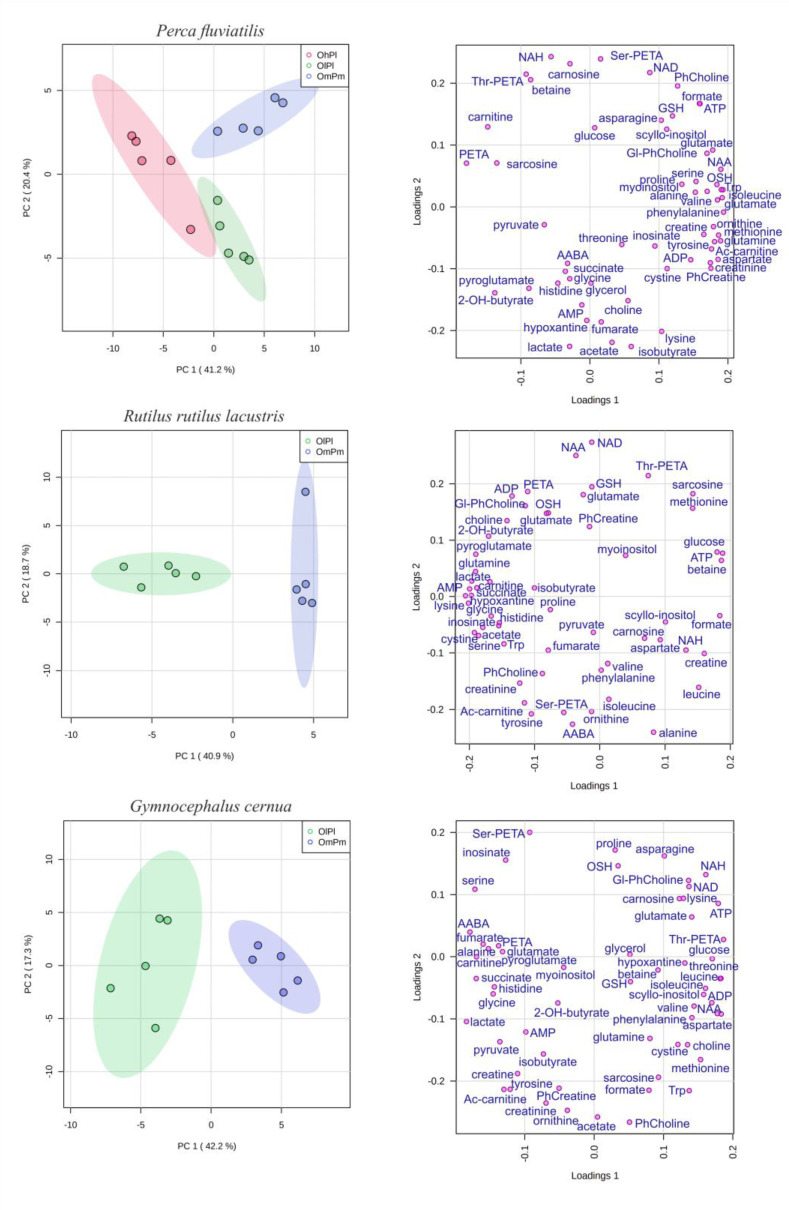
Score (**left**) and loading (**right**) plots of principal component analysis (PCA) of lens metabolomic profiles of *P. fluviatilis* (upper graphs), *R. rutilus lacustris* (middle graphs), and *G. cernua* (lower graphs) caught in the Nigiya River (green, OlPl), Lake Borovoye (red, OhPl), and the Ob Reservoir (blue, OmPm). The data are autoscaled. Colored ovals indicate 95% confidence regions. Variance explained by the first (PC1) and second (PC2) principal components are indicated on the axis.

The statistical analysis demonstrated a significant influence of location on the lenses’ metabolomic profiles. Figure 1 shows PCA plots for samples of *P. fluviatilis*, *R. rutilus lacustris*, and *G. cernua* lenses. For all three species, a distinct separation was observed between the samples collected at different locations. All individuals corresponding to the same basin were concentrated in the same cluster at the plot, although the fish were of different sexes and the size of the fish varied by a factor of two within each species. This indicated that the location was a more important factor than the sex and size of the fish. The metabolite concentrations in the lenses of fish from different locations were compared using a Mann–Whitney *U*-test. Table 2 shows a comparison of the metabolites between basins and indicates the metabolites with a statistically significant difference: no arrow—no difference, single arrows—*p*-value < 0.05, and double arrows—*p*-value < 0.01. The increase or decrease in the metabolite concentration is indicated by the direction of arrows (e.g., alanine “↑↑” in “Nigiya vs. Borovoye” means that alanine in the Nigiya River was higher than in Lake Borovoye with a *p*-value < 0.01).

**Figure 2:**
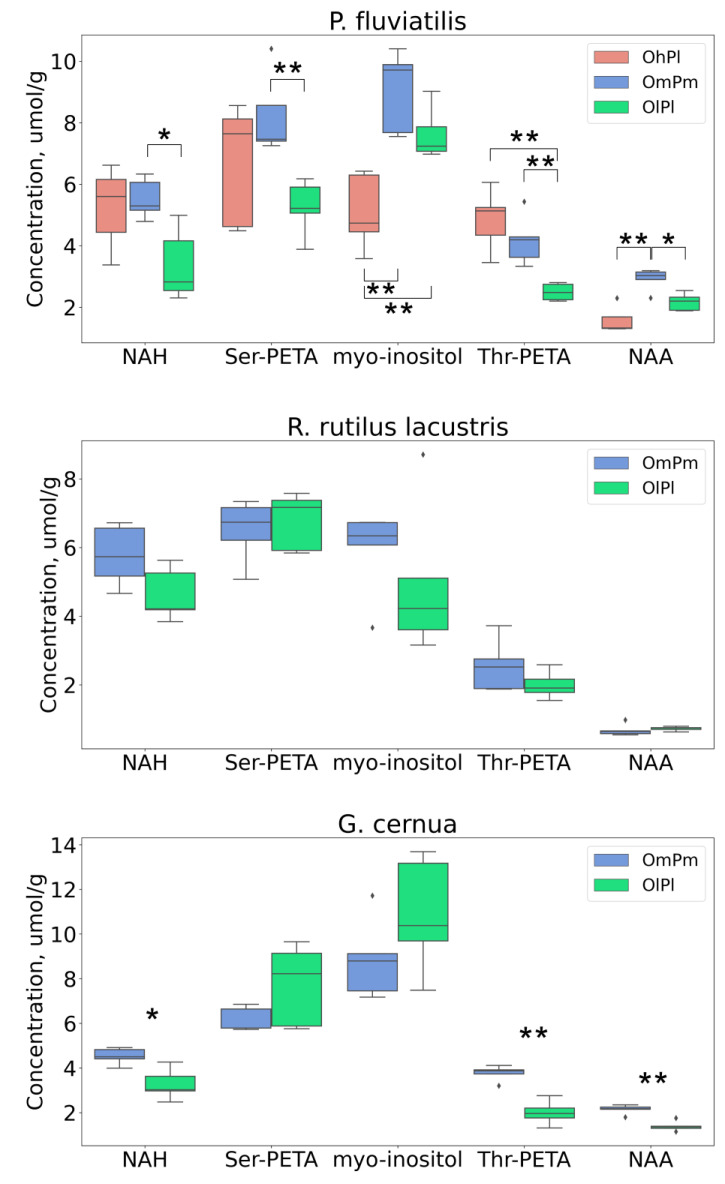
Boxplots for concentrations (in µmoles per gram of wet tissue) of five major osmolytes—NAH, ser-PETA, *myo*-inositol, thr-PETA, and NAA—in lenses of *P. fluviatilis* (upper graph), *R. rutilus lacustris* (middle graphs), and *G. cernua* (lower graph) from the Nigiya River (green, OlPl), Lake Borovoye (red, OhPl), and the Ob Reservoir (blue, OmPm). One star * indicate statistically significant difference between groups with *p*-value < 0.05, two stars ** *p* < 0.01. Rhombs indicate outliers.

We previously showed that the fish lens contains five major osmolytes—*myo*-inositol, NAH, NAA, Thr-PETA, and Ser-PETA [23,26]—and that the concentrations of these compounds in the lens undergo seasonal variations. The most significant changes observed during the winter were a decrease in NAH concentration accompanied by an increase in the *myo*-inositol level [26]. Figure 2 shows the levels of osmolytes in the fish lenses and demonstrates that the ratio NAH/*myo*-inositol was the highest for the basin with the highest LDO (Lake Borovoye, OhPl) and the lowest for the Nigiya River (OlPl), in which the LDO was low. Therefore, we assumed that the LDO is one of the main factors that regulates the composition of osmolytes in the lenses of freshwater fish.

The level of the antioxidant OSH was previously found to drop significantly during the winter: in the lenses of *R. rutilus lacustris* and pike-perch (*Sander lucioperca*), its concentration in late autumn was higher than that in early spring by factors of 4 and 2, respectively [26]. However, in the present work, we did not find a correlation between the LDO and OSH level: in *P. fluviatilis* lenses from Lake Borovoye (OhPl), the OSH concentration was 1.5–2 times lower than that from the Nigiya River (OlPl) and the Ob Reservoir (OmPm). In lenses of *R. rutilus lacustris* and *G. cernua*, there were no statistically significant differences between OSH levels for the Nigiya River and the Ob Reservoir. It was very likely that the major factor that caused the decrease in the OSH concentration in the fish lenses during the winter was a low feeding activity rather than a LDO decrease: an insufficient histidine supply resulted in the deceleration of the OSH synthesis. The elevated levels of OSH in the fish lenses from the Nigiya River and the Ob Reservoir as compared to Lake Borovoye probably indicated that the clean water and high LDO may have decreased the stimulation of OSH production. A similar conclusion could be drawn for another intracellular antioxidant, GSH, the level of which was the lowest in the lenses of *P. fluviatilis* from Lake Borovoye. However, due to the high scatter of OSH and GSH concentrations in the fish lenses (Table S3), the differences in these compounds between different locations in most cases was not statistically significant (Table 2).

**Figure 3:**
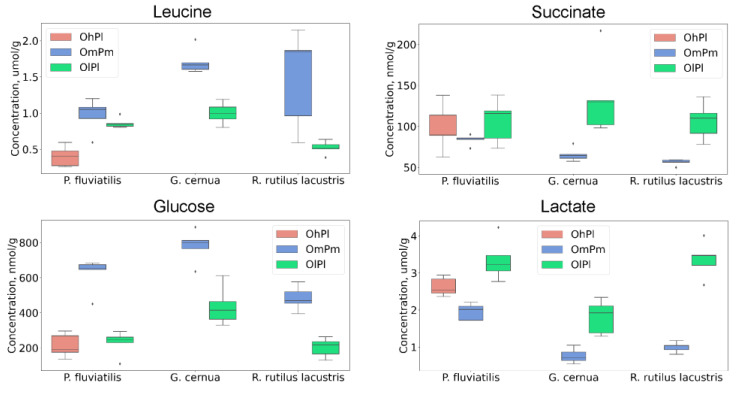
Boxplots for concentrations of leucine, succinate, glucose, and lactate in lenses of *P. fluviatilis*, *R. rutilus lacustris*, and *G. cernua* from the Nigiya River (green, OlPl), Lake Borovoye (red, OhPl), and the Ob Reservoir (blue, OmPm). Rhombs indicate outliers.

Ecological conditions affected the energy metabolic pathways. The concentration of glucose in the lenses of fish from the Nigiya River (OlPl) was significantly lower than that from the Ob Reservoir (OmPm) and Borovoye (OhPl), and the level of lactate was significantly higher (Figure 3). Lenses of fish from the Nigiya River also contain enhanced amounts of acids from the TCA cycle (fumarate and succinate) and of the final metabolic product acetate. Most likely, these effects at least partly corresponded to the low LDO in the Nigiya River.

The location also influenced the amino acid composition (branched-chain amino acids in particular). Figure 3 demonstrates that the levels of leucine in the lenses of fish from the Ob Reservoir (OmPm) were higher than those from the Nigiya River (OlPl) and Lake Borovoye (OhPl). The main difference between the Ob Reservoir and the taiga basins (Nigiya River and Lake Borovoye) was the enhanced anthropogenic pollution; the elevated concentrations of branched-chain amino acids could be attributed to the organism’s response to the elevated water acidity. This conclusion was supported by the data related to the other fish species of *R. rutilus lacustris* and *G. cernua* (Figure 3). The levels of the aromatic amino acids phenylalanine, tryptophan, and tyrosine in the lenses of *P. fluviatilis* from Lake Borovoye were 2–3 times lower than those in the two other basins; however, the obtained experimental data were not sufficient to determine the origin of this effect—a high LDO or a low pollution level—in Lake Borovoye.

Other features of the metabolomic composition of the fish lenses include enhanced levels of sarcosine and phosphoethanolamine in the lenses of *P. fluviatilis* from Lake Borovoye (OhPl) in comparison with those from the other locations. The fish lenses from the Ob Reservoir (OmPm) featured an enhanced concentration of NAD and a reduced level of AMP, although these differences were not always statistically significant.

## 4. Discussion

The present study was aimed at the evaluation of the influence of ecological factors on the metabolomic composition of the lenses of freshwater fish. The results obtained in this work indicated that even moderate variations in ecological conditions gave rise to significant changes in the metabolomic profiles of the fish lenses. We compared the metabolomic compositions of the lenses of fish from three locations with similar climates and at approximately the same time (the middle of March). According to our analysis, the water bodies differed mostly in the LDO and in contamination by anthropogenic pollutants. The LDO in the basins varied in a moderate range from 6.8 ± 0.3 mg/L (Nigiya River) to 13.1 ± 0.3 mg/L (Lake Borovoye); the water in the Ob Reservoir was moderately polluted by CO_2_ emissions, which resulted in a low pH level (Table 1). However, distinct differences were found between the metabolomic profiles of fish lenses belonging to the same species. Importantly, similar metabolomic changes were observed for various fish species including predatory (*P. fluviatilis*), herbivorous (*R. rutilus lacustris*), and omnivorous (*G. cernua*) fish. Therefore, we assumed that the observed metabolomic changes were common and that under extreme ecological conditions (very low LDO and strong water contamination with industrial waste), similar metabolomic signs will manifest themselves but at a higher scale.

The obtained data showed that the LDO in water is an extremely important factor that affects the metabolomic composition of fish tissues. The major difference between the Nigiya River and Lake Borovoye was that in the Nigiya, the LDO was twice as low as in Lake Borovoye this difference caused strong changes in the lenses’ metabolomic compositions. Table 2 shows that statistically significant (*p* < 0.05) increase in metabolite concentration in the lenses of fish from the Nigiya River as compared to Lake Borovoye were found for 21 metabolites; among these, the most significant differences (*p* <0.01) corresponded to 14 compounds. Statistically significant decrease (*p* < 0.05) were observed for five metabolites; among these, the most significant differences (*p* <0.01) were found for betaine, carnitine, Thr-PETA, and phosphoethanolamine. The correlations between the LDO and the abundances of several metabolites are shown in Figure S2. Earlier, the effects of hypoxia (low oxygen level) and anoxia (complete lack of oxygen) were studied for several aquatic species including invertebrates [9,12,36,37] and fish [8,10,11]. The authors of all of these studies agreed that the most pronounced changes in the tissue metabolomic composition under hypoxic stress corresponded to energy metabolism. However, for different species and tissues, these changes may vary significantly. For example, an ATP decrease was observed in the muscles of hypoxic red abalone (*Haliotis rufescens*) [38] and anoxic common carp (*Cyprinus carpio*) [10], but in the muscle of hypoxic *Cyprinus carpio* [10], the ATP level increased. The glutamate level increased in the muscle of red swamp crayfish (*Procambarus clarkii)* [12] and *Cyprinus carpio* [10] and in the liver of anemic catfish (*Ictalurus punctatus*) [8], but decreased in the muscle and gills of *Haliotis rufescens* [38]. A similar discrepancy was found for other metabolites including phosphocreatine, branched-chain amino acids, and compounds from the TCA cycle. Most likely, this indicated that hypoxic stress may cause different metabolic responses in different aquatic species and even in different tissues of the same species. Moreover, the observed metabolomic changes varied with time [10,11,12]. In this sense, the study of the metabolomic composition of lenses presents some advantages over other tissues: the lens is an anatomically isolated tissue that is connected with blood via the aqueous humor. Inside the lens, the metabolic activity is minimal, and the lens metabolomic profile reflects metabolomic changes that are averaged in time not only in the lens itself, but also throughout the entire body. According to the data presented in Table 2 and Table S3, the indicators of hypoxic stress in the lenses of *P. fluviatilis* might have been the increase in alanine, aspartate, glutamate, glutamine, leucine, phenylalanine, serine, valine, creatine, lactate, *myo*-inositol, and hypoxanthine; as well as the decrease in betaine, carnitine, sarcosine, Thr-PETA, GSH, and phosphoethanolamine.

It is very likely that the osmolyte NAH is also a biomarker of the oxygen level: its concentrations in the lenses of all fishes from the Nigiya River (OlPl) were lower than those in fish lenses from other locations, although these differences did not always reach a statistically significant level. Earlier [26], it was found that the level of NAH in the lenses of freshwater fish significantly decreased during the winter. The data obtained in the present work clearly indicated (Figure 2) a correlation between the LDO and the concentration of NAH in the fish lenses.

In a recent paper [8], the kidney and liver metabolomes of anemic catfish (*Ictalurus punctatus*) were compared with those of healthy ones. Significant changes were observed for the metabolites corresponding to cellular energy generation. In particular, the levels of lactate, creatine, and acetate in the anemic fish increased, and the concentrations of glucose decreased. This was in good agreement with our data: the highest levels of lactate, creatine, and acetate for all three fish species were found in the lenses of fish from the Nigiya River (low LDO).

The influence of water pollutants on the metabolic processes and metabolomic state of different tissues of fish and other aquatic species was studied in a number of previous works covered in a recent review [31]. Most often, amino acids and compounds related to the energy metabolism were considered as potential biomarkers of metabolic effects of metals, metalloids, pesticides, and other pollutants. Unfortunately, the published data on metabolic responses to the presence of toxic contaminants in water are even more controversial than the data related to the reduced oxygen level. For example, statistically significant changes in the level of branched-chain amino acids in the presence of chemical stressors in [31] were mentioned 52 times; however, in 27 cases this level increased, and in 25 cases it decreased. The level of ATP increased in 32 cases and decreased in 15 cases, and so on. These contradictions should be attributed to the following factors: (1) different types of pollutants may cause different metabolic responses in the same species; (2) the effect of the same stressor may differ for different species and for different tissues of the same species; (3) the effect of a stressor may vary with time; and (4) other environmental factors may induce significant variations in the tissue metabolomic composition that mask the effect of the stressor under study.

In the present work, the comparison of the metabolomic profiles of *P. fluviatilis* lenses from Lake Borovoye (OhPl) and the Ob Reservoir (OmPm) reflected the influence of moderate water pollution in the Ob Reservoir by CO_2_ emissions that caused the increase in the water acidity. All other factors were mostly similar: the sampling was performed in regions with similar climatic conditions at approximately the same time; the water temperature and LDO were similar as well. The comparison of the metabolomic data for the *P. fluviatilis* lenses from Lake Borovoye and the Ob Reservoir indicated that water pollution resulted in a statistically significant increase in the concentrations of 10 compounds and decrease in the levels of five metabolites (Table 2).

Table 3 presents a brief summary of the results obtained in the present work that shows the metabolomic changes induced in the fish lenses by two stressors—a reduced LDO and water pollution caused by CO_2_ emissions. It is important to note that for several metabolites, these stressors caused a similar response—such as increase in the levels of branched-chain amino acids, alanine, glutamate, and creatine; and decrease in sarcosine and phosphoethanolamine. This indicated that different stressors may affect the same metabolic pathways and cause similar metabolomic changes. At the same time, increase in the concentrations of lactate, *myo*-inositol, and hypoxanthine, as well as the decrease in NAH and Thr-PETA, can be considered as indicators specific to a reduced oxygen level; while increase in asparagine, glucose, and NAD and decrease in lactate and AMP should be attributed to the influence of anthropogenic water pollution.

**Figure 4:**
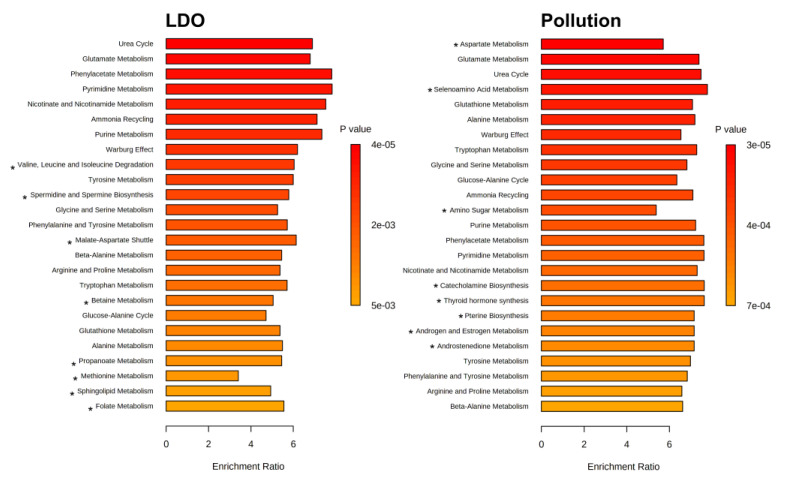
Metabolite sets enrichment analysis (MSEA) based on the comparison of metabolite concentrations in lenses of *P. fluviatilis*. Left panel: fish from lake Borovoye and from river Nigiya (influence of LDO). Right panel: fish from lake Borovoye and from Ob reservoir (influence of anthropogenic water pollution). Stars * indicate pathways different for two stressors, reduced LDO and water pollution.

The Metabolite Set Enrichment Analysis (MSEA) confirmed this statement. Figure 4 demonstrates changes in the metabolic pathways in *P. fluviatilis* lenses induced by a reduced LDO (comparison Borovoye–Nigiya) and anthropogenic pollution (comparison Borovoye–Ob Reservoir). Most of the affected pathways were common for both stressors, but approximately 30 % of the pathways (marked by stars in Figure 4) were different. According to the MSEA results depicted in Figure 4, the LDO-specific changes corresponded largely to the “Valine, Leucine and Isoleucine Degradation” and “Spermidine and Spermine Biosynthesis” pathways, while the anthropogenic water pollution selectively affected “Aspartate Metabolism” and “Selenoamino Acid Metabolism”.

It should be noted that the data obtained in the present work were the results of a field study, which had some intrinsic limitations as compared to laboratory studies. In particular, it was difficult to achieve conditions in which only one or two parameters of the environment differed while all the others remained the same. The major variables examined in the present study were the LDO and water pollution caused by CO_2_ emissions; however, other factors also could have influenced the lens metabolomic composition of the fish under study. For example, the three locations used in the study represented a river, a lake, and an artificial reservoir, and the lifestyles of fish in these basins may differ. Although the climatic conditions at all three locations were similar, the Ob Reservoir is situated approximately 500 km away from Nigiya and Borovoye, which also may have affected the fish metabolomics. For more reliable determination of biomarkers that indicate the influence of a reduced LDO and water pollution on fish metabolomics and fish health, similar studies should be performed using a greater number of locations, fish species, and physicochemical and biochemical properties. Measurements of the LDO’s influence on the fish metabolome performed under laboratory conditions would also be very useful and interesting. The important point is that the obtained metabolomic data were quantitative: we reported the values of absolute concentrations of metabolites in a tissue. These data can be directly re-used, including in data mining, with the addition of new samples or groups, in new comparisons, and so on.

## 5. Conclusions

The results obtained in the present work indicated that the anatomically isolated eye lens is a rather convenient tissue for studying the impact of ecological factors (LDO and pollution) on the metabolic state of aquatic animals. We found that two typical stressors for aquatic animals—a reduced LDO and anthropogenic water pollution—caused a largely similar metabolic response in the fish lenses. This response showed itself as increase in the concentrations of several amino acids including alanine, aspartate, glutamate, phenylalanine, branched-chain amino acids, and creatine; as well as decrease in sarcosine and phosphoethanolamine. However, distinct differences between two stressors were observed as well. In particular, the composition of the major lens osmolytes (*myo*-inositol, NAH, NAA, Thr-PETA, and Ser-PETA) depended mostly on the oxygen level, while variations in AMP (decrease) and NAD (increase) corresponded to the water pollution. A reduced LDO caused an increase in lactate, while anthropogenic water pollution caused a decrease. It is very likely that some other compounds, primarily antioxidants and energy metabolites, will also be markers of stresses induced by a reduced LDO and water pollution. However, studies performed at a larger scale (a larger number of samples in each group, a wider range of species under study, and a larger variation in stressors) are needed to establish statistically significant correlations between the concentrations of these metabolites and ecological factors.

## Figures and Tables

**Table 1:** Biochemical analysis of water samples from the Nigiya River, Lake Borovoye, and the Ob Reservoir. All data (except pH) are given in units of mg/L.

	Property	River Nigiya	Lake Borovoye	Ob Reservoir	Reference Range ^3^
1.	pH	6.8	7.5	5.3	6.0–9.0
2.	Total mineralization	125	118	<50	1500
3.	Total hardness	1.8	2.4	<0.1	10.0
4.	Ammonium ion	3.10	<0.05	0.230	2.0
5.	Iron total	0.6	<0.05	<0.05	0.3
6.	Cadmium	<0.0001	<0.0001	<0.0001	0.001
7.	Magnesium	5.40	6.40	0.080	50
8.	Manganese	<0.001	<0.001	0.0180	0.1
9.	Copper	<0.001	<0.001	<0.001	1.0
10.	Molybdenum	<0.001	<0.001	<0.001	0.07
11.	Arsenic	0.0050	<0.005	<0.005	0.01
12.	Nickel	0.0130	0.0130	0.0150	0.02
13.	Nitrate	0.13	0.32	2.23	45.0
14.	Nitrite	0.12	0.13	0.11	3.0
15.	Lead	<0.001	<0.001	<0.001	0.01
16.	Sulfate	<10.0	12.2	<10.0	500.0
17.	Chloride	<10.0	<10.0	<10.0	350.0
18.	Chrom total	<0.001	<0.001	<0.001	0.05
19.	COD ^1^	43.0	20.0	16.0	30.0
20.	BOD ^2^	18.90	8.80	7.2	4.0
21.	Suspended substances	58.4	<3.0	<3.0	-
22.	Petroleum product	0.031	0.025	0.027	0.1
23.	Bicarbonate	135.1	135.1	143.9	-
24.	Phenol	0.012	0.009	0.014	0.001
25.	Permanganate oxidizability	13.8	2.1	2.5	7.0
26.	Mercury	<0.0001	<0.0001	<0.0001	0.0005
27.	Phosphate	<0.05	<0.05	<0.05	3.5
28.	Cobalt	<0.001	<0.001	<0.001	0.1
29.	Zinc	<0.005	<0.005	<0.005	5.0
30.	LDO	6.8	13.1	9.7	-

^1^ Chemical oxygen demand; ^2^ biological oxygen demand; ^3^ values of maximum permissible concentrations for surface waters.

**Table 2:** Comparison of metabolite concentrations in lenses of *P. fluviatilis, R. rutilus lacustris*, and *G. cernua* between basins. Statistically significant difference: no arrow—no difference, single arrows—*p*-value < 0.05, and double arrows—*p*-value < 0.01. Increase or decrease in the metabolite concentration is indicated by the direction of arrows (e.g., alanine “↑↑” in “Nigiya vs. Borovoye” means that alanine in Nigiya was higher than in Borovoye with *p*-value < 0.01).

Metabolite	*P. fluviatilis*			*R. rutilus lacustris*	*G. cernua*
	Nigiya vs. Borovoye	Ob Reservoir vs. Borovoye	Nigiya vs. Ob Reservoir	Nigiya vs. Ob Reservoir	Nigiya vs. Ob Reservoir
Proteinogenic amino acids					
Alanine	↑↑	↑↑			↑↑
Asparagine		↑	↓↓		
Aspartate	↑↑	↑↑			↓↓
Glutamate	↑	↑↑		↑↑	
Glutamine	↑↑	↑↑			
Glycine				↑↑	↑
Histidine					
Isoleucine	↑↑	↑		↓	↓
Leucine	↑↑	↑	↑	↑↑	↓↓
Lysine	↑			↓	
Methionine	↑↑	↑↑			↓
Phenylalanine	↑	↑		↑↑	↓↓
Serine	↑↑	↑			↑↑
Threonine					↓↓
Valine	↑	↑			
Other amino acids					
Betaine	↓↓		↓↓	↓↓	
Carnitine	↓↓	↓↓	↓	↑↑	↑
Carnosine			↓		
Creatine	↑↑	↑↑		↓↓	
Sarcosine	↓	↓		↓↓	
Organic acids					
2-OH-butyrate		↓↓	↑↑	↑	
Acetate				↑↑	
α-Aminobutyrate					↑↑
Formate		↑↑	↓↓	↓↓	
Fumarate			↑		↑↑
Isobutyrate	↑↑		↑↑		
Lactate	↑	↓↓	↑↑	↑↑	↑↑
Pyroglutamate		↓↓	↑↑	↑↑	↑↑
Succinate				↑↑	↑↑
Osmolytes					
*myo*-Inositol	↑↑	↑↑			
NAA		↑↑	↓		↓↓
NAH			↓		↓
Ser-PETA			↓↓		
Thr-PETA	↓↓		↓↓		↓↓
Antioxidants					
Cystine					
				↑↑	
Alcohols, amines, and sugars					
Choline				↑	
Glucose		↑	↓↓	↓↓	↓↓
Gl-PhCholine		↑↑			↓↓
PhCholine		↓↓	↓↓		
Phosphoethanolamine	↓↓	↑			↑
*scyllo*-Inositol				↓↓	↓
Glycerol				↑↑	
Nitrogenous bases, nucleotides, and nucleosides					
ADP					↓
AMP				↑↑	
ATP		↑↑	↓	↓↓	↓
Creatinine	↑↑	↑↑			
Hypoxanthine			↑↑	↑↑	
Inosinate				↑	↑
NAD		↑	↓↓		

**Table 3:** Changes in the metabolite concentrations in lenses of freshwater fish under influences of reduced LDO and anthropogenic water pollution caused by CO_2_ emissions. Arrows indicate the direction of changes.

Metabolite	Stressor	
	Reduced LDO	Water Pollution
Proteinogenic amino acids		
Alanine	↑	↑
Asparagine		↑
Aspartate	↑	↑
Glutamate	↑	↑
Glutamine	↑	↑
Leucine	↑	↑
Phenylalanine	↑	↑
Serine	↑	↑
Valine	↑	↑
Other amino acids		
Betaine	↓	
Carnitine	↓	
Creatine	↑	↑
Sarcosine	↓	↓
Organic acids		
Formate		↑
Lactate	↑	↓
Osmolytes		
*myo*-Inositol	↑	
NAA		↑
NAH	↓	
Thr-PETA	↓	
Alcohols, amines, and sugars		
Glucose		↑
Phosphoethanolamine	↓	↓
Nitrogenous bases, nucleotides, and nucleosides		
AMP		↓
NAD		↑

## Data Availability

The NMR spectra and metabolite concentrations are available in the Animal Metabolite Database repository under the Experiment IDs 70, 95, and 119 (https://amdb.online, accessed on 22 June 2021). All other data are available from the corresponding author upon request.

## Supplementary Materials

The following supporting information can be downloaded at: www.mdpi.com/xxx/s1/. Supplementary Table S1: Characterization of fish lenses used in this study. Supplementary Table S2: Chemical shifts and multiplicities of NMR signals of metabolites identified in this work. Supplementary Table S3: Concentrations of metabolites in lenses of *P. fluviatilis*, *R. rutilus lacustris*, and *G. cernua* (in nmoles per gram of wet tissue). Supplementary Figure S1: Map showing locations of the fish sample collection. Supplementary Figure S2: Correlations between LDO level and abundances of metabolites in the lens of *P. fluviatilis*.
