# Supplementary material for: Influence of Ecological Factors on the Metabolomic Composition of Fish Lenses
Source: Biology (Basel). 2022 Nov 25;11(12):1709. doi: 10.3390/biology11121709 (PMC9774591; doi:10.3390/biology11121709)

Supplementary Information

for

Influence of Ecological Factors on the Metabolomic Composition of Fish Lenses

Yuri P. Tsentalovich*, Ekaterina A. Zelentsova, Ekaterina D. Savina, Vadim V. Yanshole, Renad Z. Sagdeev

International Tomography Center SB RAS, Institutskaya 3a, Novosibirsk 630090, Russia

* Correspondence: yura@tomo.nsc.ru

Table of content

Supplementary Table S1. Characterization of fish lenses used in this study.

Supplementary Table S2. Chemical shifts and multiplicities of NMR signals of metabolites identified in this work.

Supplementary Table S3. Concentrations of metabolites in lenses P. fluviatilis, R. rutilus lacustris, and G. cernua (in nmoles per gram of wet tissue).

Supplementary Figure S1. Map showing locations of the fish sample collection.

Supplementary Figure S2. Correlations between LDO level and abundances of metabolites in the lens of *P. fluviatilis.*

Supplementary Table S1. Characterization of fish lenses used in this study.

Sample Number	Fish Type	Date of catching	Location	Lens weight, mg
Group 1				
OHL1	*P. fluviatilis*	14.03.2019	Nigiya	61
OHL2	*P. fluviatilis*	14.03.2019	Nigiya	60
OHL3	*P. fluviatilis*	14.03.2019	Nigiya	150
OHL4	*P. fluviatilis*	14.03.2019	Nigiya	97
OHL5	*P. fluviatilis*	14.03.2019	Nigiya	56
Group 2				
OSL1	*P. fluviatilis*	11.03.2019	Borovoye	135
OSL2	*P. fluviatilis*	11.03.2019	Borovoye	140
OSL3	*P. fluviatilis*	11.03.2019	Borovoye	135
OSL4	*P. fluviatilis*	11.03.2019	Borovoye	85
OSL5	*P. fluviatilis*	11.03.2019	Borovoe	86
Group 3				
OOL1	*P. fluviatilis*	11.03.2019	Ob reservoir	89
OOL2	*P. fluviatilis*	11.03.2019	Ob reservoir	88
OOL3	*P. fluviatilis*	11.03.2019	Ob reservoir	82
OOL4	*P. fluviatilis*	11.03.2019	Ob reservoir	87
OOL5	*P. fluviatilis*	11.03.2019	Ob reservoir	113

Group 4				
CHL1	*R. rutilus lacustris*	14.03.2019	Nigiya	69
CHL2	*R. rutilus lacustris*	14.03.2019	Nigiya	86
CHL3	*R. rutilus lacustris*	14.03.2019	Nigiya	112
CHL4	*R. rutilus lacustris*	14.03.2019	Nigiya	103
CHL5	*R. rutilus lacustris*	14.03.2019	Nigiya	94
Group 5				
COL1	*R. rutilus lacustris*	09.03.2019	Ob reservoir	91
COL2	*R. rutilus lacustris*	09.03.2019	Ob reservoir	102
COL3	*R. rutilus lacustris*	09.03.2019	Ob reservoir	88
COL4	*R. rutilus lacustris*	09.03.2019	Ob reservoir	85
COL5	*R. rutilus lacustris*	09.03.2019	Ob reservoir	79
Group 6				
EHL1	*G. cernua*	14.03.2019	Nigiya	138
EHL2	*G. cernua*	14.03.2019	Nigiya	118
EHL3	*G. cernua*	14.03.2019	Nigiya	152
EHL4	*G. cernua*	14.03.2019	Nigiya	96
EHL5	*G. cernua*	14.03.2019	Nigiya	85
Group 7				
EOL1	*G. cernua*	09.03.2019	Ob reservoir	118
EOL2	*G. cernua*	09.03.2019	Ob reservoir	103
EOL3	*G. cernua*	09.03.2019	Ob reservoir	125
EOL4	*G. cernua*	09.03.2019	Ob reservoir	82
EOL5	*G. cernua*	09.03.2019	Ob reservoir	118

Supplementary Table S2. Chemical shifts and multiplicities of NMR signals of metabolites identified in this work.

Metabolite	Functional group	Chemical shift	Multiplicity
Proteinogenic amino acids			
Alanine	CH_3	1.47	Doublet
Asparagine	CH	2.84	Doublet of Doublets
	CH	2.94	Doublet of Doublets
Aspartate	CH	2.67	Doublet of Doublets
	CH	2.80	Doublet of Doublets
	CH	3.89	Doublet of Doublets
Glutamate	CH_2	2.34	Multiplet
	CH	3.75	Multiplet
Glutamine	CH_2	2.13	Multiplet
	CH_2	2.44	Multiplet
	CH	3.76	Multiplet
Glycine	CH_2	3.54	Singlet
Histidine	CH	7.06	Singlet
	CH	7.80	Singlet
Isoleucine	CH_3	0.93	Triplet
	CH_3	1.00	Doublet
Leucine	$(CH_3)_2$	0.95	Doublet of Doublets
Lysine	CH_2	3.01	Triplet
	CH	3.74	Triplet
Methionine	CH_3	2.12	Singlet
	CH_2	2.62	Triplet
	CH	3.85	Doublet of Doublets
Proline	CH	4.12	Doublet of Doublets
Serine	CH	3.83	Doublet of Doublets
	CH_2	3.93	Doublet of Doublets
Threonine	CH_3	1.32	Doublet
	CH	3.57	Doublet
	CH	4.24	Multiplet
Tryptophan	CH	7.27	Multiplet
	CH	7.31	Doublet of Triplets
	CH	7.53	Doublet of Triplets
	CH	7.73	Multiplet
Tyrosine	$(CH)_2$	6.89	Multiplet
	$(CH)_2$	7.18	Multiplet
Valine	CH_3	0.98	Doublet
	CH_3	1.03	Doublet
Other amino acids			
Ac-Carnitine	$(CH_3)_3$	3.18	Singlet
Betaine	$(CH_3)_3$	3.25	Singlet
	CH_2	3.89	Singlet
Carnitine	$(CH_3)_3$	3.21	Singlet
Carnosine	CH	7.04	Singlet
	CH	8.00	Singlet
Creatine	CH_3	3.02	Singlet
	CH_2	3.92	Singlet
Ornithine	CH	3.77	Triplet
	CH_2	3.04	Triplet
Phosphocreatine	CH_3	3.03	Singlet
	CH_2	3.94	Singlet
Sarcosine	CH_3	2.73	Singlet

Organic acids			
2-OH-butyrate	CH_3	0.89	Triplet
Acetate	CH_3	1.90	Singlet
α-Aminobutyrate	CH_3	0.97	Triplet
Formate	CH	8.44	Singlet
Fumarate	CH_2	6.50	Singlet
Isobutyrate	$(CH_3)_2$	1.06	Doublet
Lactate	CH_3<br>CH	1.31<br>4.09	Doublet<br>Quartet
Pyroglutamate	CH_2<br>CH	2.39<br>4.16	Multiplet<br>Doublet of Doublets
Pyruvate	CH_3	2.36	Singlet
Succinate	$(CH_2)_2$	2.39	Singlet
Osmolytes			
myo-Inositol	CH<br>$(CH)_2$<br>$(CH)_2$<br>CH	3.26<br>3.52<br>3.61<br>4.05	Triplet<br>Doublet of Doublets<br>Triplet<br>Triplet
NAA	CH_3<br>CH<br>CH	2.01<br>2.48<br>2.68	Singlet<br>Doublet of Doublets<br>Doublet of Doublets
NAH	CH_3<br>CH<br>CH<br>CH<br>CH<br>CH	1.97<br>3.00<br>3.16<br>4.44<br>7.06<br>8.06	Singlet<br>Doublet of Doublets<br>Doublet of Doublets<br>Doublet of Doublets<br>Doublet<br>Doublet
Ser-PETA	CH_2<br>CH<br>CH_2<br>CH<br>CH	3.27<br>3.98<br>4.08<br>4.23<br>4.30	Multiplet<br>Multiplet<br>Multiplet<br>Multiplet<br>Multiplet
Thr-PETA	CH_3<br>CH_2<br>CH<br>CH_2<br>CH	1.45<br>3.25<br>3.74<br>4.04<br>4.82	Doublet<br>Triplet<br>Doublet<br>Doublet<br>Multiplet
Antioxidant			
GSH	-<br>-<br>-<br>CH<br>-	2.55<br>2.94<br>3.77<br>4.56<br>2.15	Multiplet<br>Multiplet<br>Multiplet<br>Doublet of Doublets<br>Quartet
OSH	CH_3<br>CH	3.71<br>8.23	Doublet<br>Quartet
Cystine	CH_2<br>CH_2<br>CH_2	3.18<br>3.38<br>4.11	Doublet of Doublets<br>Doublet of Doublets<br>Doublet of Doublets
Alcohols. amines. sugars			
Choline	$(CH_3)_3$	3.19	Singlet
Glucose	CH<br>$(CH)_2$<br>CH<br>CH	3.23<br>3.39<br>3.47<br>3.52	Doublet of Doublets<br>Multiplet<br>Multiplet<br>Doublet of Doublets

	$(CH)_2$	3.71	Multiplet
	CH	3.83	Multiplet
	CH	3.87	Doublet of Doublets
	CH	4.63	Doublet
	CH	5.22	Doublet
Gl-PhCholine	$(CH_3)_3$	3.22	Singlet
PhCholine	$(CH_3)_3$	3.21	Singlet
	CH_2	3.58	Multiplet
	CH_2	4.15	Multiplet
Phosphoethanolamine	CH_2	3.21	Multiplet
	CH_2	3.98	Multiplet
scyllo-Inositol	$(CH)_6$	3.33	Singlet
Glycerol	CH_2	3.55	Doublet of Doublets
	CH_2	3.64	Doublet of Doublets
Nitrogenous bases. nucleotides. nucleosides			
ADP	CH	6.14	Doublet
	CH	8.26	Singlet
	CH	8.53	Singlet
AMP	CH	4.50	Multiplet
	CH	6.14	Doublet
	CH	8.25	Singlet
	CH	8.60	Singlet
ATP	CH	6.14	Doublet
	CH	8.26	Singlet
	CH	8.52	Singlet
Creatinine	CH_3	3.03	Singlet
	CH_2	4.05	Singlet
Hypoxanthine	CH	8.18	Singlet
	CH	8.20	Singlet
Inosinate	CH	6.13	Doublet
	CH	8.21	Singlet
	CH	8.56	Singlet
NAD	CH	6.03	Doublet
	CH	6.07	Doublet
	CH	8.16	Singlet
	CH	8.19	Multiplet
	CH	8.42	Singlet
	CH	8.82	Multiplet
	CH	9.14	Doublet
	CH	9.33	Singlet

Supplementary Table S3. Concentrations of metabolites in lenses *P. fluviatilis, R. rutilus lacustris*, and *G. cernua* (in nmoles per gram of wet tissue).

Metabolite	*P. fluviatilis*			*R. rutilus lacustris*		*G. cernua*	
	**river Nigiya** (OlPl)	**Ob reservoir** (OmPm)	**lake Borovoye** (OhPl)	**river Nigiya** (OlPl)	**Ob reservoir** (OmPm)	**river Nigiya** (OlPl)	**Ob reservoir** (OmPm)
**Proteinogenic amino acids**							
**Alanine**	3500 ± 700	3700 ± 300	2230 ± 250	2300 ± 400	3000 ± 1300	2900 ±300	2390 ± 190
**Asparagine**	340 ± 100	610 ± 110	380 ± 140	ND	ND	30 ± 30	90 ± 70
**Aspartate**	770 ± 200	730 ± 80	260 ± 120	260 ± 70	380 ± 130	650 ± 140	1090 ± 170
**Glutamate**	2780 ± 270	3400 ± 500	2130 ± 270	1800 ± 280	1760 ± 290	2900 ± 400	3400 ± 400
**Glutamine**	2700 ± 500	2600 ± 500	790 ± 290	6400 ± 900	4200 ± 700	1000 ± 300	1230 ± 210
**Glycine**	390 ± 100	260 ± 60	380 ± 230	450 ± 130	260 ± 90	233 ± 19	170 ± 30
**Histidine**	760 ± 120	642 ± 24	840 ± 260	580 ± 130	340 ± 70	420 ± 90	352 ± 21
**Isoleucine**	238 ± 21	290 ± 80	120 ± 50	72 ± 16	74 ± 26	130 ± 50	230 ± 50
**Leucine**	860 ± 70	970 ± 230	400 ± 140	520 ± 90	1500 ± 700	1000 ± 150	1710 ± 180
**Lysine**	178 ± 27	135 ± 27	121 ± 23	240 ± 22	60 ± 9	58 ± 9	68 ± 10
**Methionine**	520 ± 90	540 ± 200	180 ± 120	76 ± 28	220 ± 100	320 ± 100	630 ± 200
**Phenylalanine**	690 ± 170	830 ± 220	260 ± 160	300 ± 90	340 ± 130	310 ± 80	560 ± 100
**Proline**	230 ± 28	260 ± 60	210 ± 40	90 ± 40	71 ± 29	100 ± 50	112 ± 26
**Serine**	1940 ± 90	2100 ± 500	1310 ± 290	3600 ± 400	2450 ± 230	1520 ± 210	1090 ± 70
**Threonine**	1500 ± 180	1310 ± 180	1300 ± 300	760 ± 100	710 ± 130	540 ± 110	960 ± 140
**Tryptophan**	490 ± 110	630 ± 190	200 ± 110	270 ± 50	180 ± 70	160 ± 50	240 ± 50
**Tyrosine**	900 ± 300	940 ± 150	280 ± 150	620 ± 170	460 ± 210	550 ± 220	390 ± 50
**Valine**	370 ±40	440 ± 110	220 ± 100	100 ± 40	100 ± 40	170 ± 90	320 ± 90
**Other amino acids**							
**Ac-Carnitine**	23 ± 6	21 ± 5	6.1 ± 2.9	19 ± 5	14 ± 5	14 ± 4	10.1 ± 1.6
**Betaine**	42 ± 5	70 ± 15	81 ± 14	31 ± 10	131 ± 26	200 ± 40	220 ± 30
**Carnitine**	70 ± 18	110 ± 24	181 ± 29	120 ± 40	33 ± 9	44 ± 11	20 ± 7
**Carnosine**	41 ± 14	68 ± 15	60 ± 13	64 ± 12	77 ± 22	36 ± 19	55 ± 11
**Creatine**	38 ± 6	38 ± 9	13 ± 5	31 ± 6	63 ± 14	62 ± 20	47.9 ± 2.3
**Ornithine**	230 ± 80	250 ± 70	70 ± 60	150 ± 50	160 ± 50	160 ± 70	150 ± 40
**Phosphocreatine**	60 ± 21	51 ± 12	14 ± 10	46 ± 13	47 ± 10	32 ± 8	29.8 ± 2.6
**Sarcosine**	19 ± 8	21 ± 4	46 ± 29	4.0 ± 2.2	25 ± 15	9 ± 8	14 ± 3

**Organic acids**							
**2-OH-butyrate**	20 ± 6	5 ± 4	26 ± 5	12 ± 4	5 ± 3	9 ± 6	7 ± 6
**Acetate**	6800 ± 2200	4500 ± 400	5400 ± 1300	6100 ± 900	4500 ± 600	4000 ± 700	4000 ± 700
**α-Aminobutyrate**	200 ± 90	170 ± 90	210 ± 50	270 ± 110	250 ± 110	133 ± 23	60 ± 20
**Formate**	66 ± 22	190 ± 50	45 ± 11	55 ± 20	158 ± 12	132 ± 24	150 ± 30
**Fumarate**	31 ± 6	21 ± 3	25 ± 4	12.6 ± 2.2	9 ± 6	50 ± 14	26 ± 5
**Isobutyrate**	22 ± 5	10.3 ± 1.1	10 ± 5	6 ± 4	3 ± 3	4.2 ± 1.8	3.3 ± 1.1
**Lactate**	3400 ± 600	1960 ± 220	2630 ± 250	3400 ± 500	1000 ± 140	1800 ± 500	760 ± 200
**Pyroglutamate**	260 ± 70	120 ± 18	310 ± 180	300 ± 100	90 ± 50	125 ± 30	63 ± 23
**Pyruvate**	14.3 ± 2.5	14 ± 4	21 ± 7	7 ± 3	7 ± 3	9 ± 4	4.6 ± 2.4
**Succinate**	106 ± 26	84 ± 6	99 ± 29	106 ± 22	56 ± 4	140 ± 50	66 ± 8
**Osmolytes**							
***myo*-Inositol**	7600 ± 800	9100 ± 1300	5100 ± 1200	5000 ± 2200	5900 ± 1300	10900 ± 2600	8800 ± 1800
**NAA**	2180 ± 280	2900 ± 300	1600 ± 400	730 ± 60	690 ± 170	1390 ± 220	2140 ± 210
**NAH**	3400 ± 1200	5500 ± 600	5200 ± 1300	4600 ± 800	5800 ± 900	3300 ± 700	4500 ± 400
**Ser-PETA**	5200 ± 900	8200 ± 1300	6700 ± 2000	6800 ± 800	6500 ± 900	7700 ± 1800	6300 ± 500
**Thr-PETA**	2490 ± 280	4100 ± 800	5000 ± 1000	2000 ± 400	2600 ± 800	2000 ± 500	3800 ± 300
**Antioxidants**							
**GSH**	150 ± 110	340 ± 180	110 ± 70	50 ± 40	60 ± 50	30 ±13	50 ± 30
**OSH**	1400 ± 280	1800 ± 400	900 ± 300	390 ± 110	320 ± 170	400 ± 400	600 ± 400
**Cystine**	222 ± 29	210 ± 70	170 ± 40	250 ± 30	169 ± 18	141 ± 17	169 ± 25
**Alcohols, amines, sugars**							
**Choline**	280 ± 80	230 ± 60	260 ± 120	130 ± 40	70 ± 30	255 ± 27	300 ± 40
**Glucose**	230 ± 70	620 ± 100	210 ± 70	200 ± 50	480 ± 70	440 ± 110	780 ± 90
**Gl-PhCholine**	159 ± 28	240 ± 80	120 ± 60	72 ± 17	55 ± 16	90 ± 30	170 ± 50
**PhCholine**	1010 ± 190	1760 ± 290	1060 ± 170	1650 ± 270	1460 ± 190	490 ± 150	560 ± 80
**Phosphoethanolamine**	530 ± 160	560 ± 210	2200 ± 1000	1100 ± 300	700 ± 300	1100 ± 300	560 ± 230
***scyllo*-Inositol**	37 ± 16	100 ± 70	27 ± 11	16 ± 8	90 ± 100	80 ± 60	220 ± 70
**Glycerol**	150 ± 50	95 ± 29	140 ± 50	90 ± 40	0	129 ± 30	150 ± 40
**Nitrogenous bases, nucleotides, nucleosides**							

**ADP**	210 ± 30	210 ± 24	170 ± 50	258 ± 23	220 ± 40	186 ± 8	221 ± 22
**AMP**	80 ± 40	54 ± 12	90 ± 70	220 ± 40	67 ± 20	116 ± 21	91 ± 20
**ATP**	580 ± 100	900 ± 200	480 ± 90	500 ± 40	820 ± 100	480 ± 30	570 ± 30
**Creatinine**	55 ± 22	49 ± 10	15 ± 12	43 ± 13	32 ± 10	35 ± 14	30 ± 3
**Hypoxanthine**	107 ± 7	83 ± 12	100 ± 30	101 ± 23	ND	26 ± 7	33 ± 6
**Inosinate**	48 ± 5	46 ± 4	37 ± 12	130 ± 40	70 ± 23	52 ± 8	36 ± 12
**NAD**	117 ± 20	186 ± 30	144 ± 15	87 ± 7	80 ± 40	102 ± 22	128 ± 24

Supplementary Figure S1. Map showing locations of the fish sample collection.

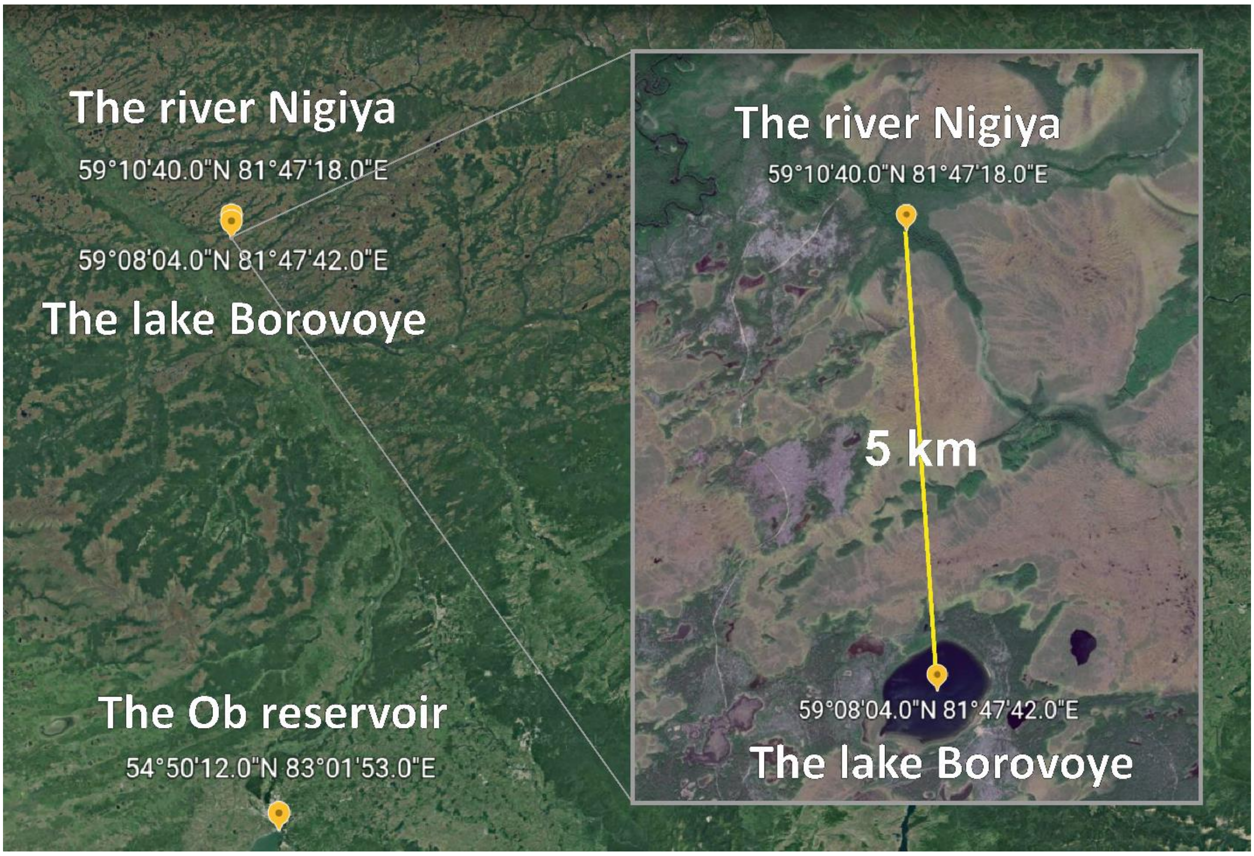

Supplementary Figure S2. Correlations between LDO level and abundances of metabolites in the lens of *P. fluviatilis*.

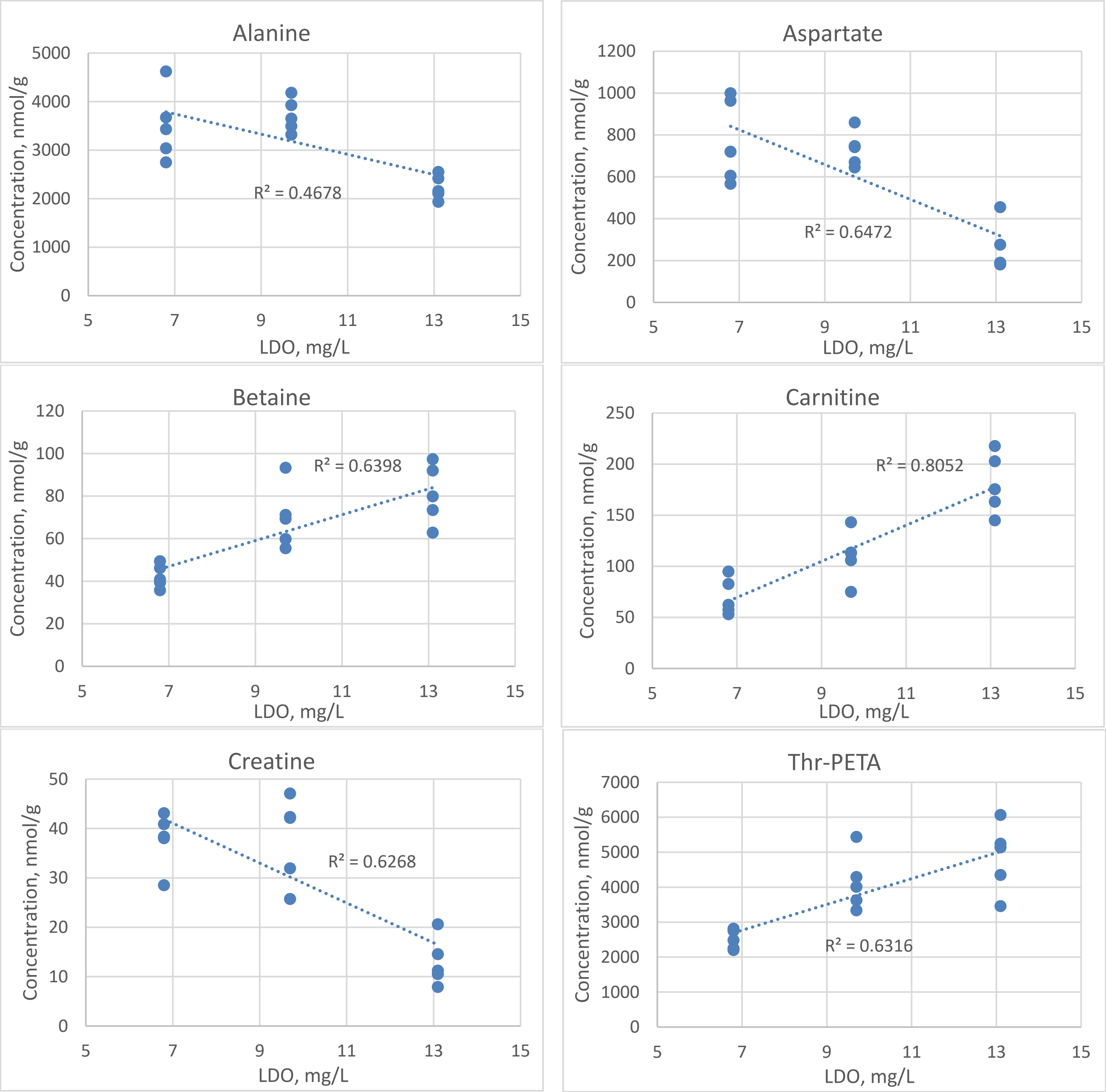

Supplement: Supplementary file 1 [file biology-11-01709-s001.zip › biology-2037272-supplementary.pdf]
